# Assessing Clinical Outcomes in Colorectal Cancer with Assays for Invasive Circulating Tumor Cells

**DOI:** 10.3390/biomedicines6020069

**Published:** 2018-06-06

**Authors:** Yue Zhang, Kevin Zarrabi, Wei Hou, Stefan Madajewicz, Minsig Choi, Stanley Zucker, Wen-Tien Chen

**Affiliations:** 1 Stony Brook Medicine, Stony Brook, NY 11794, USA; kevin.zarrabi@stonybrookmedicine.edu (K.Z.); wei.hou@stonybrookmedicine.edu (W.H.); stefanmadajewicz@yahoo.com (S.M.); minsig.choi@stonybrookmedicine.edu (M.C.); s_zucker@yahoo.com (S.Z.); wentien@vitatex.com (W.-T.C.); 2 Division of Hematology/Oncology, Department of Medicine, Stony Brook University Hospital, Stony Brook, NY 11794, USA; 3 Department of Medicine and Research, Veterans Affairs Medical Center, Northport, NY 11768, USA; 4 Vitatex Inc., 25 Health Sciences Drive, Stony Brook, NY 11790, USA

**Keywords:** circulating tumor cells, colorectal carcinoma, CAM invasion assay, phenotypic mosaics, tumor progenitor

## Abstract

Colorectal carcinoma (CRC) is the second leading cause of cancer-related mortality. The goals of this study are to evaluate the association between levels of invasive circulating tumor cells (iCTCs) with CRC outcomes and to explore the molecular characteristics of iCTCs. Peripheral blood from 93 patients with Stage I–IV CRC was obtained and assessed for the detection and characterization of iCTCs using a functional collagen-based adhesion matrix (CAM) invasion assay. Patients were followed and assessed for overall survival. Tumor cells isolated by CAM were characterized using cell culture and microarray analyses. Of 93 patients, 88 (95%) had detectable iCTCs, ranging over 0–470 iCTCs/mL. Patients with Stage I–IV disease exhibited median counts of 0.0 iCTCs/mL (*n* = 6), 13.0 iCTCs/mL (*n* = 12), 41.0 iCTCs/mL (*n* = 12), and 133.0 iCTCs/mL (*n* = 58), respectively (*p* < 0.001). Kaplan–Meier curve analysis demonstrated a significant survival benefit in patients with low iCTC counts compared with in patients with high iCTC counts (log-rank *p* < 0.001). Multivariable Cox model analysis revealed that iCTC count was an independent prognostic factor of overall survival (*p* = 0.009). Disease stage (*p* = 0.01, hazard ratio 1.66; 95% confidence interval: 1.12–2.47) and surgical intervention (*p* = 0.03, HR 0.37; 95% CI: 0.15–0.92) were also independent prognostic factors. Gene expression analysis demonstrated the expression of both endothelial and tumor progenitor cell biomarkers in iCTCs. CAM-based invasion assay shows a high detection sensitivity of iCTCs that inversely correlated with overall survival in CRC patients. Functional and gene expression analyses showed the phenotypic mosaics of iCTCs, mimicking the survival capability of circulating endothelial cells in the blood stream.

## 1. Introduction

Colorectal carcinoma (CRC) is the second leading cause of cancer-related mortality in the United States with an incidence rate of 135,430 new cases and an estimated 50,260 deaths in 2017 [1]. Patients with Stage IV disease have a 5-year survival rate under 10% [2]. Of patients with Stage II or III disease, 25–50% suffer from relapse, likely as a consequence of undetected spread of malignant cells, even after radical surgery and adjuvant therapy [3,4]. A prospective biomarker is needed to better predict disease recurrence, treatment response, and drug resistance, and to better understand the molecular mechanism of tumor cell invasion [5].

Circulating tumor cells (CTCs) have been isolated and have proven prognostic value in CRC patients [6,7,8]. We have developed a functional collagen-based adhesion matrix (CAM) enrichment assay for identifying CTCs that are positively expressing epithelial markers EpCAM and ESA/CD24 (Epi+), and for detecting the subpopulation that also expresses CAM uptake (CAM+) or invasion marker seprase and stem cell marker CD44, termed invasive CTCs (iCTCs) [9,10]. Detection, enrichment, and clinical utilities have been demonstrated in both metastatic and nonmetastatic cancers of the breast, ovary, and prostate [9,10,11,12,13,14,15]. Here, we utilized the CAM invasion assay to detect iCTCs from the blood of patients with colorectal carcinoma. Patients with biopsy-proven colorectal carcinoma were recruited. Peripheral blood samples (2 mL each assay) were collected from patients with Stage I–IV disease and were applied to the CAM system. iCTCs were quantified and the patients were followed with routine clinic exams. Herein, we have shown that iCTCs have prognosticative value in patients with CRC. Specifically, we assess the association between overall survival and the level of iCTCs. This study supports the paradigm that assessment for iCTCs may be utilized by clinicians in identifying individuals with CRC who are at high risk for aggressive disease [16]. Furthermore, we evaluated the clinical significance of iCTCs in patients receiving surgery, chemotherapy, and radiation. Microarray and cell culture analyses further demonstrated the possibility for iCTCs to be potentially used as a prognostic biomarker in CRC patients.

## 2. Materials and Methods

### 2.1. Patient and Clinical Samples

This study was performed through Stony Brook University Medical Center and the Department of Veterans Affairs Medical Center, Northport, NY, USA. The study was performed with the approval of the Institutional Review Board and the Committee on Research in Human Subjects (ID code 100593, renewed 7 November 2016). Specimens were collected from 19 October 1999 to 24 November 2015. Patients were followed and monitored for disease recurrence and mortality. The patients were staged according to the American Joint Committee on Cancer TNM system. Samples were obtained via three to twenty milliliter (mL) collections of peripheral blood (*n* = 93). Blood was collected in Vacutainer^TM^ tubes (Becton Dickinson, Franklin Lakes, NJ, USA; green top, lithium heparin as anticoagulant) and processed within four hours of collection. Two milliliter aliquots of blood were employed for quantification of iCTCs. Blood was collected in the clinic and processed in the laboratory according to the workflow below; detailed steps were described in a previous paper [14].

### 2.2. Clinical Data Collection

All clinical data and end points were collected and documented in a de-identified fashion. All data was entered and stored in a Microsoft Excel worksheet. Data was extracted from the electronic medical record of the patient’s medical charts and collected through 1 November 2016. Overall survival length was calculated from the date of blood sample collection to the date of death or most recent documented contact.

### 2.3. Cell Culture, Identification of CTCs and iCTCs, and Functional Proliferation/Invasion Assays

Cell culture, identification of CTCs and iCTCs, and proliferation/invasion assays using the functional CAM enrichment platform have been described previously [9,14]. Briefly, nuclear cells from 2 mL whole blood aliquots were seeded onto one well of a CAM-coated 6-well plate (Vita-Assay™, Vitatex Inc., Stony Brook, NY, USA) with the complete cell culture (CCC) medium and incubated in a CO_2_ incubator for 12–18 h for capture of CTCs, iCTCs, and approximately 0.1% leukocytes, collectively called CAM-avid cells. Using the experimental tumor-cell-spiked-in blood, the Vita-Assay™ enrichment platform functionally captured and enriched (up to one-million-fold) 98% of tumor cells spiked in blood—CTCs—with 0.01–1.0% purity (most co-isolating cells were leukocytes). Furthermore, the platform exhibited the unique advantage that CAM-avid cells could be identified as iCTCs after subsequent ingestion of fluorescent CAM (CAM+). Since the functional proclivities to degrade and ingest the extracellular matrix are major acquired capabilities of invasive and metastatic cells, CAM+ cells represent a unique way to identify iCTCs.

To culture iCTCs ex vivo, old media and nonadherent cells in the same plate were removed and replaced with fresh media every three days for continued culture. Proliferative and differentiative activities of iCTCs were determined by their capability to duplicate and form stem-cell-like colonies and epithelial morphology.

To identify CTCs and iCTCs, cells adhered on CAM-coated plates were collected, fixed, and stained for microscopy using anti-hematopoietic lineage (HL) antibody against CD45 (clone T29/33, DakoCytomation, Carpinteria, CA, USA); anti-epithelial pan-cytokeratins 4, 5, 6, 8, 10, 13, and 18 (CK) (clone C11, Sigma, St. Louis, MO, USA); or anti-endothelial CD31 (Clone JC/70A, NeoMarkers, Fremont, CA, USA); this was followed by red or blue color alkaline-phosphatase-anti-alkaline-phosphatase (APAAP) secondary antibodies (DakoCytomation), then by staining with fluorescein isothiocyanate (FITC)- or tetramethylrhodamine (TRITC)-conjugated anti-tumor progenitor (TP) antibodies (anti-CD44 and anti-seprase, Vitatex) or anti-epithelia (EPI) antibodies (ESA clone B29.1, Biomeda; EPCAM clone Ber-Ep4). Stained cells in suspension were mounted using a Cytospin device (StatSpin cytofuge and Filter Concentrators). Microscopic analyses were performed on a Nikon Eclipse E400 inverted fluorescence microscope equipped with a Microfire digital camera system and Image Pro Plus software. EPI + CD45- cells were identified as CTCs; EPI + CD45-CAM+ or TP+ cells were iCTCs. Microscopic counting of cells was performed by trained personnel and confirmed by a second observer.

**Figure 1:**
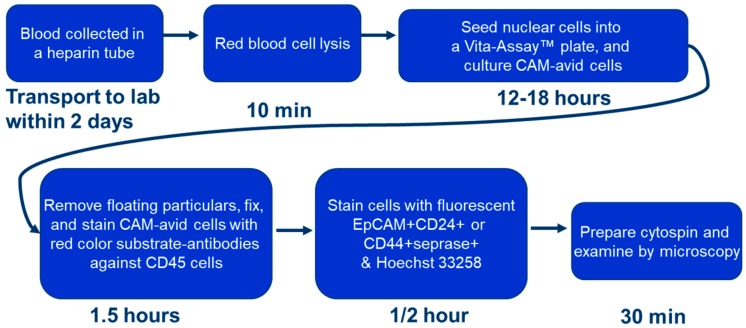
Flow chart representing the procedural steps of invasive circulating tumor cell (iCTC) isolation and quantification.: CAM indicates collagen-based adhesion matrix.

The invasive activity of iCTCs was determined by the CAM uptake assay using fluorescently labeled CAM-coated 6-well plates, as described [14]. Cells exhibiting CAM uptake (CAM+) were identified as a functional label for iCTCs. To demonstrate the acquired endothelial function by iCTCs, CAM-adherent cells were incubated with fluorescein-acetylated low-density lipoprotein (acLDL, Invitrogen, Carlsbad, CA, USA) at 37 °C in a CO_2_ incubator for three hours. Cells exhibiting acLDL uptake were identified as either circulating endothelial cells or iCTCs in CRC blood (Figure 1).

### 2.4. Microarray Data Analysis

CAM-adherent cells that were directly isolated from 1 mL of whole blood using a CAM-coated tube (Vita-Cap™, Vitatex Inc., Stony Brook, NY, USA) were used in DNA microarray analysis, as described [9]. Briefly, generation of cRNA, labeling, hybridization, and scanning of the Affymetrix high-density oligonucleotide microarray HG_U133_Plus_2 chip (containing 54,675 gene probes) were performed according to the manufacturer’s specifications (Affymetrix, Santa Clara, CA, USA). Analysis of each chip was performed using the Affymetrix Microarray Suite 5.1 Software to generate raw expression data. GeneSpring 7.2 software (Silicon Genetics, Redwood City, CA, USA) was used to assist in the statistical analysis and the selection of genes specific for CAM-enriched circulating cells.

### 2.5. Statistical Analysis

Patient data was stratified as continuous or categorical variables. Continuous variables, such as patient age and iCTC counts, were analyzed by way of medians, means, and standard errors. Due to the fact the data distribution was not normal, nonparametric statistics (Kruskal–Wallis test for multiple groups and Mann–Whitney U test for two groups) were employed. Categorical variables, such as patient gender, race, disease stage, and history of chemotherapy/radiation therapy/surgery were listed as frequencies and percentages. Categorical data was analyzed with chi-square tests.

Effects of iCTC counts on overall survival were evaluated using the Cox proportional hazards models. For multivariable analyses, iCTC and other characteristics (e.g., gender, race, chemotherapy, stage, surgery, and radiation) and their interactions were all included in the Cox model. For univariable analyses, iCTC counts were included as a sole predictor in the Cox model. To determine an optimal cutoff point of iCTCs, which can best differentiate the survival curve by high- and low-iCTC groups, the Cox model was fitted iteratively using all possible cutoff points. The cutoff point with the best model fitting index akaike information criterion (AIC) (lowest value) was selected as the optimal point. The patients were categorized into the high- and low-iCTC groups using the optimal cutoff point. The survival curve for each group was estimated using the Kaplan–Meier method. Log-rank tests were used to assess survival differences between patients who had high iCTC counts versus low iCTC counts. iCTC detection was defined as the presence of at least one detectable iCTC. Results with two-tailed *p*-values less than 0.05 were considered statistically significant. All analyses were performed using SAS v9.4 (the SAS Institute, Cary, NC, USA).

## 3. Results

### 3.1. Patient Characteristics

Ninety-three patients (M/F: 63/30; median age: 62.0 years, range: 35–82 years) with colon and rectal cancers had samples collected and were analyzed in this study. Of the 93 total patients, 88 patients (95%) had detectable iCTCs and iCTC positives correlated with disease status: Stage I, 6 patients (7%); Stage II, 12 patients (14%); Stage III, 12 patients (14%); and Stage IV, 58 patients (65%) (Table 1). A total of 64 (69%) patients were undergoing treatment for tumors in the colon and 29 (31%) for tumors in the rectum. Patients received various treatments: chemotherapy, 71 (81%); surgical resection, 74 (84%); and radiation therapy, 30 (34%). There was no significant correlation between iCTCs and patient gender, race, disease stage, or age.

### 3.2. Clinical Characteristics of iCTCs: Stage and Survival

**Figure 2:**
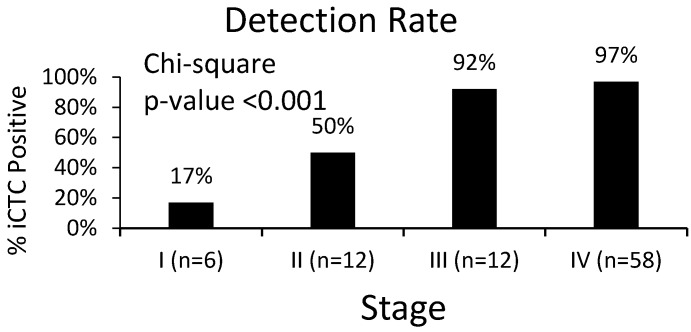
iCTC detection rate by disease stage. Percentage of patients within each stage group and their detectable iCTC rates. iCTC detection varied considerably between Stage III/IV disease and Stage I/II disease (*p*-value < 0.001).

**Figure 3:**
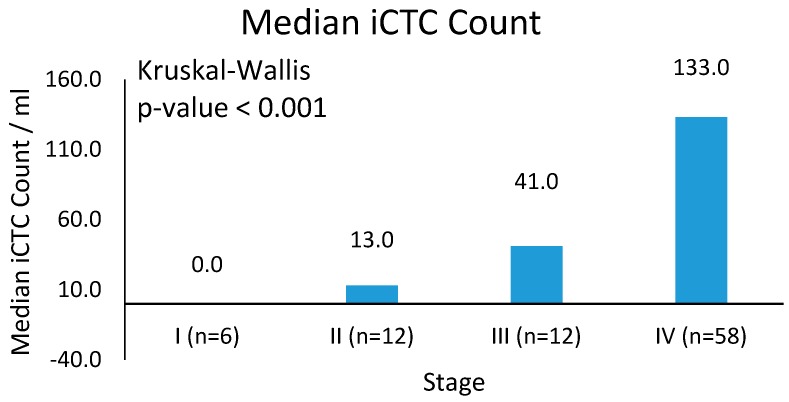
Mean iCTC counts. Median iCTCs by stage with SD. Median iCTC counts differ significantly between Stage IV disease and Stage I–III disease (*p*-value < 0.001).

A baseline of 5.0 iCTCs/mL was used as a predetermined cutoff for iCTC positivity with readings below 5.0 iCTCs/mL considered undetectable levels (Table 2). Data comparing iCTCs and CTCs were available for 31 patients, in which 38% to 72% of CTCs overlapped with iCTCs. However, CTCs as identified using Epi+ markers were high in patients with benign disease (85%, *n* = 30), suggesting low specificity. Analyses were performed to establish a correlation between iCTC measurements and clinical staging. Overall, 95% (*n* = 88) of patients had detectable iCTCs at the time of evaluation. There is a significant correlation between disease stage and iCTCs: 92% (*n* = 12) of patients with Stage III disease and 97% (*n* = 58) of patients with Stage IV disease had detectable iCTCs. This is opposed to only 17% (*n* = 6) and 50% (*n* = 12) of patients with Stages I and II disease, respectively (Figure 2, *p*-value < 0.001). Analysis of the median iCTC count as a function of disease stage demonstrated significance (Figure 3; Table 3). Median iCTC counts of 0.0 iCTCs/mL (*n* = 6), 13.0 iCTCs/mL (*n* = 12), 41.0 iCTCs/mL (*n* = 12), and 133.0 iCTCs/mL (*n* = 58) were observed for Stages I, II, III, and IV disease, respectively (Figure 3; *p*-value < 0.001); mean iCTC counts of 8.3 iCTCs/mL (*n* = 6), 35.8 iCTCs/mL (*n* = 12), 65.9 iCTCs/mL (*n* = 12), and 144.8 iCTCs/mL (*n* = 58) were observed for Stages I, II, III, and IV disease, respectively.

**Figure 4:**
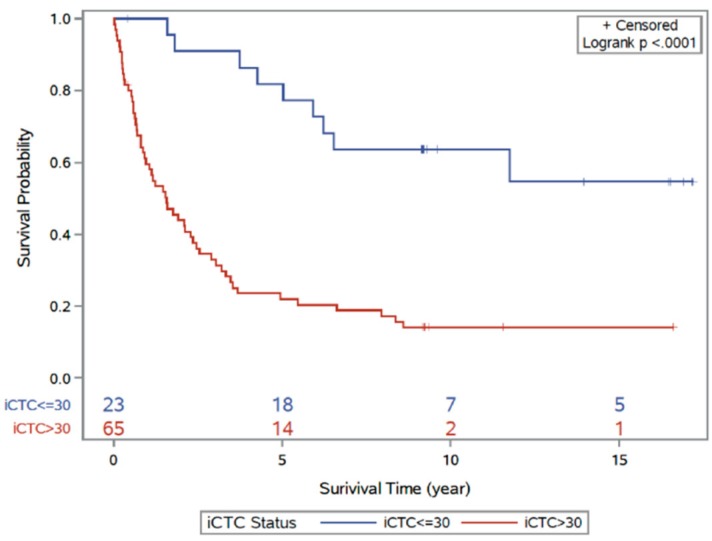
Kaplan–Meier curve analysis for disease outcome according to high-iCTC-count groups and low-iCTC-count groups using optimal iCTC cutoff point (≤30 iCTCs/mL vs. >30 iCTCs/mL). The two survival curves are significantly different (log-rank *p*-value < 0.001). The red line indicates the high iCTC count group; blue line indicates the low iCTC count group.

Patients were followed to assess the correlation of iCTC counts and patient survival. Mean follow-up time was 71.7 months (range 1.0–143.2 months). At the conclusion of the study, 28% (*n* = 26) of patients were living and 72% of patients (*n* = 67) were deceased. Patients were stratified by an arbitrary cutoff of 30 iCTCs/mL for survival analysis and survival times were compared between patients with high iCTC counts (>30 iCTCs/mL) and low iCTC counts (≤30 iCTCs/mL; lowest AIC = 506.8). A significant decrease in survival time was observed in patients who had iCTC counts greater than 30 iCTCs/mL (Figure 4; log-rank *p*-value < 0.001). The hazard ratio of survival increases by 5% for every 10 iCTCs/mL (hazard ratio 1.05, 95% CI: 1.03–1.07, *p* < 0.001). When evaluated by cancer type, iCTC counts correlate significantly with survival in colon cancer (hazard ratio: 1.06, 95% CI: 1.03–1.09, *p* < 0.0001) but with a trend toward significance in rectal cancer (hazard ratio: 1.03, 96% CI: 1.00–1.07, *p* = 0.06).

A multivariable analysis of the survival data from this patient cohort was performed to establish correlations between iCTC counts and patient characteristics (e.g., cancer type, stage, gender, chemotherapy, surgery, radiation, and age); iCTC counts, stage, and surgical treatment remained significant with adjustments for other covariates (Table 4). These results indicate that iCTC count is an independent prognostic factor for distant metastases (hazard ratio = 1.66, 95% CI: 1.12–2.47, *p* = 0.01). As expected, surgical intervention as assessed by iCTC count was associated with improved outcome (hazard ratio = 0.37, 95% CI: 0.15–0.92, *p* = 0.03). As iCTC counts increase by 10 units, the hazard ratio increases by 4% (Table 4; 95% CI 1.12–2.47, *p* = 0.01). If the disease status increases by one stage, the hazard ratio increases by 66% (95% CI: 1.12–2.47, *p* = 0.01). For patients who had undergone surgery, the hazard ratio is reduced by 63% (*p* = 0.03).

### 3.3. Molecular and Functional Phenotyping of iCTCs in CRC Patients

iCTCs represent a subpopulation of CTCs that exhibit the phenotype of a metastasis-initiating cell in blood [17], including proliferation and tumor differentiation, invasion, progenitor cell potency, and survival capability in the blood stream.

**Figure 5:**
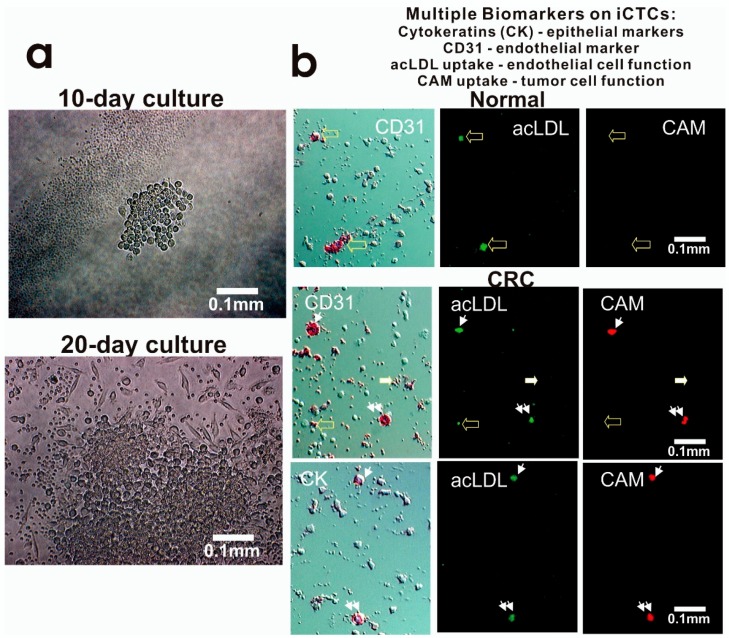
Proliferative and invasive activities and expression of multiple cell lineage markers of iCTCs in blood of CRC patients. (**a**) Proliferation and differentiation of iCTCs into epithelial colonies ex vivo. CAM-enriched cells were cultured on the CAM scaffold for ten days and twenty days. Live cells were photographed under phase contrast microscopy. Tumor cells grew in clusters with large epithelioid cells but hematologic cells (solitary small cells and platelet-like cell fragments seen in the lower image) decreased in number and became not evident; (**b**) iCTCs express epithelial and endothelial biomarkers as well as display epithelial and endothelial functions. Cell multipotency of iCTCs was verified in single cells using expression of epithelial cytokeratins (CK) and endothelial CD31, acLDL uptake of endothelial function, and CAM uptake of tumor progenitor cell function. Background cells that were not labeled with antibody staining were leukocytes and platelets co-isolated with iCTCs. (**Upper**) panel: circulating endothelial cells in normal blood were seen to be CD31+ acLDL uptake+ but CAM uptake−. (**Middle**) panel: iCTCs in blood of a Stage IV CRC patient were seen to show CD31+ acLDL uptake+ CAM uptake+ (indicated by small arrows and double small arrows). However, circulating endothelial cells and platelets were seen to be CD31+ acLDL uptake± CAM uptake− (indicated by large solid and open arrows). (**Lower**) panel: iCTCs in blood of a Stage IV CRC patient were seen to show CK+ acLDL uptake+ CAM uptake+ (indicated by small arrows and double small arrows).

To determine the proliferative and functional activities of iCTCs, we captured iCTCs in CRC blood using Vita-Assay™ plates and cultured the cells on the same CAM substrata. We successfully cultured iCTCs for up to four weeks in blood of 56 out of 61 CRC patients (92%), a result similar to those in both metastatic and nonmetastatic cancers of the breast, ovary, and prostate [9,10,11,12,13,14,15]. The number and size of cells increased over time in culture, resulting in sizeable colonies within 10 days (Figure 5a) and large-spread cells with epithelial morphology after 20 days (Figure 5a), whereas co-purified hematopoietic cells were observed in reduced number over time (Figure 5a). The growth rate of iCTCs was estimated by counting numbers of cells in colonies formed in Day 5 cultures, which were composed of 16–32 iCTCs, suggesting a doubling time of about 34–42 h for iCTCs. These ex vivo results indicate that the iCTC phenotype includes the ability to propagate and progress to tumor cell morphology.

To examine whether iCTCs surviving in the blood stream acquire the phenotype of circulating endothelial cells, cells captured in the CAM-coated wells were immuno-stained with antibodies against epithelial CK and endothelial CD31, and underwent functional analyses using CAM uptake for invasive tumor cells as described [14] and acLDL uptake for circulating endothelial cells as described [18]. iCTCs showed tumor-cell-like morphology, were CK+ and CD31+, and showed both acLDL and CAM uptakes (Figure 5b). This finding shows that iCTCs acquire the phenotypic characteristics of circulating endothelial cells, such as the expression of the endothelial lineage marker and the endothelial function to adopt for their survival in the blood stream and extravasation.

**Figure 6:**
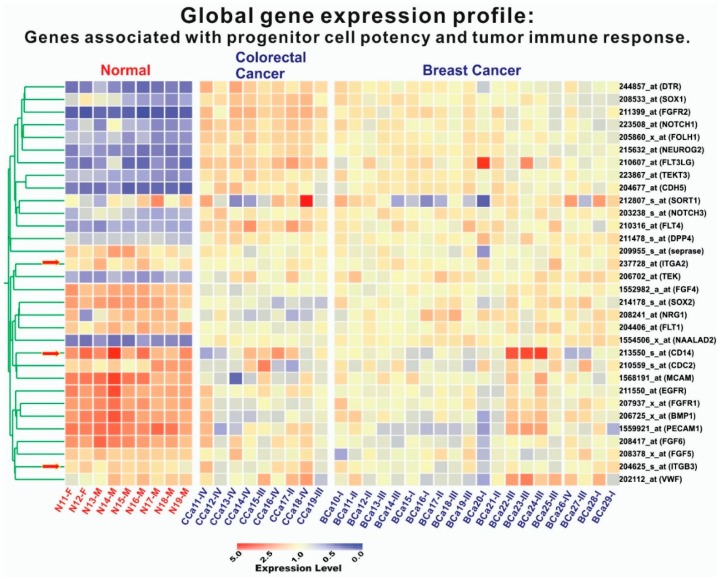
Expression of tumor-progenitor-associated genes in iCTCs isolated by CAM from blood of patients with CRC and breast cancer. Global gene expression profiling of circulating cells isolated by Vita-Cap™ from 9 healthy subjects, 9 CRC patients, and 20 patients with breast cancer. Columns represent catalogues of cell samples analyzed: circulating Normal (N) cells isolated from healthy donors with suffix M for Male and F for Female; CCa are circulating Colorectal Cancer (CCa) cells and BCa are circulating Breast Cancer (BCa) cells with suffixes I–IV being stages of the disease. Colorgram depicts high (red) and low (blue) relative levels of gene expression. Red arrows indicate the three internal control genes that exhibited no difference between normal and cancer cell samples.

Based on the assumption that subsets of endothelial cell genes are upregulated in iCTCs, global gene expression profiling of cells isolated by CAM from the blood of healthy subjects and of CRC and breast cancer patients were conducted using Affymetrix HG_U133_Plus_2 chips containing 54,675 gene probes (Figure 6). Genes related to multiple cell lineage–progenitor potency (*DTR*, *SOX1*, *FGFR2*, *NOTCH1*, *FOLH1*, *NEUROG2*, *FLT3LG*, *TEKT3*, *CDH5*, *FLT4*, *TEK*) and tumor immune response (*DPP4/CD26*) were upregulated in 9 CRC samples and 20 breast cancer samples, but other endothelial genes (*FGF4*, *SOX2*, *NRG1*, *FLT1*, *CDC2*, *MCAM*, *EGFR*, *FGFR1*, *BMP1*, *PECAM1*, *FGF6*, *FGF5*, *VWF*) were not (Figure 5). In addition, four cytokeratin genes (*KRT8*, *KRT16*, *KRT17*, and *KRT19*) and eight tumor-associated genes (*TERT*, *MUC16/M17S2/CA125*, *CD44*, *TWIST1*, *TACSTD1/EpCAM/CD326/ESA/HEA125/GA733*, *DPP4/CD26*, *ESR1*, and *PGR*), were upregulated in CRC and breast cancer samples, as described previously [9]. The expression data strongly suggest that iCTCs express a subset of endothelial markers, in addition to their own tumor progenitor markers.

## 4. Discussion

CTCs have been demonstrated to have potential clinical validity in a variety of different cancers [19]. CTC enumeration is a potential method for early detection, treatment monitoring, and response assessment in colorectal cancer patients [20,21]. Unfortunately, the relationship between CTC counts and clinicopathological parameters remains unknown [22]. There is a paucity of data regarding CTC counts and clinical outcomes. It has not been used in clinical practice to date [23].

In this study, we utilized an iCTC detection system, which assesses for CAM adhesive and invasive properties in conjunction with tumor progenitor marker expression in patients with colorectal cancer. We showed that microscopic examination of CAM-avid cells detected iCTCs with a high sensitivity, i.e., 88 of 93 patients (95%) had detectable iCTCs, ranging over 0–470 iCTCs/mL. In addition, we found that patients with Stages I, II, III, and IV disease exhibited median iCTC counts of 0.0 iCTCs/mL, 13.0 iCTCs/mL, 41.0 iCTCs/mL, and 133.0 iCTCs/mL, respectively (*p*-value < 0.001), suggesting that our iCTC detection method may have the highest sensitivity compared with previous methods [20,21,22,23], which detected 1 CTC/7.5 mL blood from fractions of patients with Stage IV disease. A possible explanation for the more than 10-fold detection sensitivity and specific detection in early stages of disease from our method is that CAM is an artificial extracellular matrix mimic that exerts powerful attractive force for solid-tissue-derived cells themselves including CTCs and iCTCs circulating in blood. Other methods rely on devices with specific filtration, microfluidic dynamics, or antibody-coated microcarriers to extract CTCs from whole blood. Other methods, therefore, might miss a portion of CTCs that are small in size, lack specified physical properties, or are low in expression of needed cell surface antigens.

Our data supports the paradigm whereby iCTC count is a promising biomarker with diagnostic and prognostic value. In our cohort of 93 patients, those with advanced disease harbored significantly higher iCTCs. We found that surgical intervention affected iCTCs in colorectal cancer (HR 0.37), in contrast to previous studies that focused on CTC count correlation with chemotherapy [24]. Analysis of our patients revealed a significant survival benefit in those with low iCTC counts. iCTC counts were not significantly associated with gender, age, or race. Due to limitations in sample size, data was not stratified to account for differences in patients with colon or rectal cancers.

CTCs provide a unique source of tumor-derived material for molecular analysis [24]. The majority of the genes identified were involved in cell motility, cell adhesion, chemokine activity, signal transduction, and cell proliferation in a previous study using cDNA from CTCs [25]. We performed global gene expression profiling from the isolated iCTCs and found upregulation of multiple cell lineage–progenitor potency (*DTR*, *SOX1*, *FGFR2*, *NOTCH1*, *FOLH1*, *NEUROG2*, *FLT3LG*, *TEKT3*, *CDH5*, *FLT4*, *TEK*), tumor immune response (*DPP4/CD26*), cytokeratin genes (*KRT8*, *KRT16*, *KRT17*, and *KRT19*), and tumor-associated genes (*TERT*, *MUC16/M17S2/CA125*, *CD44*, *TWIST1*, *TACSTD1/EpCAM/CD326/ESA/HEA125/GA733*, *DPP4/CD26*, *ESR1*, and *PGR*). In this study, gene expression profiling supports the hypothesis that iCTCs are a subpopulation of CTCs that exhibit the phenotype of a metastasis-initiating cell in blood [17], including proliferation and tumor differentiation, invasion, progenitor cell potency, and tumor immune response. This pattern is consistent with a previous report of cDNA from CTCs in CRC using microarray analysis [25].

In this study, the detected iCTC counts correlated with tumor burden and disease staging, suggesting that iCTCs have the potential to be a monitoring and prognostic biomarker in CRC. Also, we have validated use of the CAM method in detection of iCTCs in patients with CRC and provided groundwork for future prospective studies. For example, treatment monitoring for early recurrence of disease has yet to be evaluated using iCTCs in the CRC patient cohort. Pearl et al. has reported that increases in iCTCs predated relapse of epithelial ovarian cancer earlier than cancer antigen 125 (CA-125) monitoring [12]. Wang et al. demonstrated that postoperative detection of CTCs detects disease relapse early while carcinoembryonic antigen (CEA) levels are within normal limits. CTC levels were found to have a six-month lead time over CEA levels with relation to disease recurrence [26]. These studies exemplify the promising role of CTCs as a surrogate tumor marker. However, while the small sample size of 93 patients only in this study allowed for proof-of-principle demonstrations and hypothesis generation of iCTCs in colorectal cancer, a larger cohort study is warranted to determine the prognostic relevance of iCTCs in CRC patients. Moreover, that there are no studies to date investigating a head-to-head comparison between CAM assay and the traditional anti-EpCAM approach, although such studies may be technically challenging [27].

In summary, we provide data on iCTCs enriched through a functional CAM assay in the CRC patient cohort. iCTCs are readily enriched and detectable with the approach. We found that iCTC levels correlate clinically with disease stage and tumor burden, providing promising evidence for the prognosticative value of iCTCs. iCTCs may serve as a surrogate biomarker for colorectal tumors. Further studies are warranted to determine if iCTCs may have a role in early detection of disease recurrence, drug response testing, and genomic studies. Our findings highlight the clinical significance of iCTCs in CRC and suggest a role for iCTC quantification in disease diagnosis, treatment monitoring, and post-treatment surveillance.

## Figures and Tables

**Table 1:** Characteristics by cancer type.

		Total (*n* = 88 *)
Male		63 (72%)
Race	Caucasian	70 (80%)
	African American	5 (6%)
	Asian	2 (2%)
	Hispanic	3 (3%)
	Unknown	8 (9%)
Stage	1	6 (7%)
	2	12 (14%)
	3	12 (14%)
	4	58 (65%)
Chemotherapy		71 (81%)
Surgery		74 (84%)
Radiation		30 (34%)

Patient baseline clinical–pathological characteristics. * Of 93 total patients, 88 patients had detectable iCTCs.

**Table 2:** CTC detectability by stage.

Stage	CTC < 5	CTC ≥ 5	CTC ≥ 5%
I (*n* = 6)	5	1	17%
II (*n* = 12)	6	6	50%
III (*n* = 12)	1	11	92%
IV (*n* = 58)	2	56	97%

Distribution of iCTC counts over different stage groups.

**Table 3:** iCTC median, mean and SD.

Stage	iCTC Median	iCTC Mean	iCTC Standard Error
I (*n* = 6)	0.0	8.3	8.3
II (*n* = 12)	13.0	35.8	13.5
III (*n* = 12)	41.0	65.9	23.1
IV (*n* = 58)	133.0	144.8	13.8

**Table 4:** Hazard ratio (HR) for stage and surgery.

Factor	Univariable Model		Multivariable Model	
	Hazard Ratio (95% CI)	*p* Value	Hazard Ratio (95% CI)	*p* Value
iCTCs	1.05 (1.03–1.07)	<0.0001	1.04 (1.01–1.06)	0.009
Stage	1.89 (1.37–2.61)	0.0001	1.66 (1.12–2.47)	0.01
Surgery	0.17 (0.08–0.36)	<0.0001	0.37 (0.15–0.92)	0.03

Cox regression multivariate analysis of prognostic factors for colorectal carcinoma.

## Abbreviations

Progression-free survival	PFS
Overall survival	OS
Colorectal carcinoma	CRC
Circulating tumor cells	CTCs
Epithelial cell adhesion molecule	EpCAM
Collagen adhesion matrix	CAM
Epithelial markers	Epi+
Hazard ratio	HR
Confidence interval	CI
Akaike information criterion	AIC
Invasive CTCs	iCTCs
Carcinoembryonic antigen	CEA
